# Application of Wavelet Feature Extraction and Artificial Neural Networks for Improving the Performance of Gas–Liquid Two-Phase Flow Meters Used in Oil and Petrochemical Industries

**DOI:** 10.3390/polym13213647

**Published:** 2021-10-23

**Authors:** Siavash Hosseini, Osman Taylan, Mona Abusurrah, Thangarajah Akilan, Ehsan Nazemi, Ehsan Eftekhari-Zadeh, Farheen Bano, Gholam Hossein Roshani

**Affiliations:** 1 Department of Software Engineering, Lakehead University, Thunder Bay, ON P7B 5E1, Canada; hoseini.svh@gmail.com (S.H.); takilan@lakeheadu.ca (T.A.); 2 Department of Industrial Engineering, Faculty of Engineering, King Abdulaziz University, P.O. Box 80204, Jeddah 11451, Saudi Arabia; otaylan@kau.edu.sa (O.T.); fbano@kau.edu.sa (F.B.); 3 Department of Management and Information Systems, College of Business Administration, Taibah University, P.O. Box 344, Medina 42353, Saudi Arabia; ma-busurrah@taibahu.edu.sa; 4 Imec-Vision Lab, Department of Physics, University of Antwerp, 2610 Antwerp, Belgium; ehsan.nazemi@uantwerpen.be; 5 Institute of Optics and Quantum Electronics, Friedrich Schiller University Jena, Max-Wien-Platz 1, 07743 Jena, Germany; 6 Electrical Engineering Department, Kermanshah University of Technology, Kermanshah 6715685420, Iran; hosseinroshani@kut.ac.ir

**Keywords:** wavelet, feature extraction, two-phase, flow measurement

## Abstract

Measuring fluid characteristics is of high importance in various industries such as the polymer, petroleum, and petrochemical industries, etc. Flow regime classification and void fraction measurement are essential for predicting the performance of many systems. The efficiency of multiphase flow meters strongly depends on the flow parameters. In this study, MCNP (Monte Carlo N-Particle) code was employed to simulate annular, stratified, and homogeneous regimes. In this approach, two detectors (NaI) were utilized to detect the emitted photons from a cesium-137 source. The registered signals of both detectors were decomposed using a discrete wavelet transform (DWT). Following this, the low-frequency (approximation) and high-frequency (detail) components of the signals were calculated. Finally, various features of the approximation signals were extracted, using the average value, kurtosis, standard deviation (STD), and root mean square (RMS). The extracted features were thoroughly analyzed to find those features which could classify the flow regimes and be utilized as the inputs to a network for improving the efficiency of flow meters. Two different networks were implemented for flow regime classification and void fraction prediction. In the current study, using the wavelet transform and feature extraction approach, the considered flow regimes were classified correctly, and the void fraction percentages were calculated with a mean relative error (MRE) of 0.4%. Although the system presented in this study is proposed for measuring the characteristics of petroleum fluids, it can be easily used for other types of fluids such as polymeric fluids.

## 1. Introduction

Information on flow regimes is used to increase the performance of flow meters [1]. Regime recognition and void fraction measurement are important issues in many industrial applications [2]. A wide variety of methods have been proposed for determining these parameters. The gamma-ray attenuation technique is the most accurate of these [3,4]. Over the past several years, many studies have been performed to enhance the accuracy of this method. Abro and co-authors [5] advanced a new methodology based on multi-beam gamma-ray densitometry for flow regime classification and void fraction calculation. The dual modality densitometry technique was studied by Jiang et al. [6] for regime classification in a two-phase flow using a radial basis function (RBF) neural network. Abro et al. carried out a study investigating the efficiency of single-beam and multi-beam gamma-ray densitometry in predicting the void fraction in two-phase flow systems [7]. Based on the presented results, the multi-beam gamma-ray technique showed better performance than the single-beam technique. Nazemi and co-authors performed research into void fraction measurement that was independent of density changes in the liquid phase, by employing a dual modality densitometry technique [3].

Roshani et al. [1] studied flow regime identification in a two-phase flow structure utilizing the dual modality densitometry method. For regime identification and void fraction measurement, three features were extracted. The extracted features were considered as the inputs of an ANN. Nazemi et al. utilized a method to investigate the optimum position of the scattering detector involving two features for achieving greater accuracy [8]. In this study, an RBF neural network was used to accomplish the investigations. This strategy enabled them to calculate volumetric percentages autonomously of the density changes. A new approach was investigated by Peyvandi and co-workers that aimed to calculate the void fraction in some specific conditions where only one side of the pipe is accessible [9]. Nazemi et al. carried out research into two-phase flow regimes for volume fraction calculations autonomously of the various types of regimes in the pipe, using the gamma-ray attenuation technique [10]. In all the previously mentioned studies, three or more detectors were employed, but in the latter study, the gamma-ray attenuation technique made it possible to use two detectors in the structure. Subsequently, a new study was performed to identify three flow regimes in a specific condition. Roshani et al. used one source and one detector to classify three flow regimes in their system, but only two of them were identified correctly [11].

Roshani et al. studied void fraction measurements independently of the type of flow regimes using a dual-energy broad beam technique. In this context, several features were extracted from the signals from the detector to use in network inputs [12]. Hanus et al. studied time-domain feature extraction to recognize flow regimes by employing different types of neural networks [13]. Sattari and co-workers used time-domain feature extraction for estimating volumetric percentages and identifying flow regimes. In this study, void fraction percentages were calculated with an MRE of 5.32 [14].

In the current study, a system including one source (cesium-137) and two detectors (NaI) was used to register the emitted photons passing through a pipe. Three main regimes consisting of homogeneous, stratified, and annular regimes with void fractions in the range of 5–90% were considered and simulated using MCNP code. The signals from both detectors were decomposed using DWT into approximation and detail components. In order to achieve regime identification and void fraction measurement, two methods for extracting features to utilize as the inputs of ANNs were analyzed.

In this paper, by utilizing an optimized structure and taking advantage of the wavelet transform, the types of regimes and volume fraction percentages in a two-phase flow were obtained precisely. In this study, the features of the signals were extracted using a wavelet transform. Then, various feature extraction methods were applied to the signals and finally, using the defined SA parameter, the best features with the best separation ability were selected. Following this, the features were applied to the networks, and the results demonstrated the precision and correctness of the presented solution. This method reduced the rate of errors in terms of the void fraction.

## 2. Simulated Structure

MCNP code, which is a powerful tool for modeling radiation-based multiphase flow meters [15,16,17,18,19,20,21,22,23,24], was used in this study to model the measuring system. In the simulations, homogeneous, stratified, and annular regimes were considered in the structure. Simulations were accomplished for void fractions ranging from 5% to 90% for all the aforementioned regimes. Gas oil and air were defined as the liquid and gas phases, respectively. It is worth noting that radiation-based multiphase flow meters are independent of the chemical characteristics of the materials inside the pipe that the radiation passes through. In other words, it makes no difference whether the radiation passes through a polymeric fluid, water, gas oil, or any other type of fluid. Therefore, instead of gas oil, which is used as an example liquid inside the pipe in this study, there could be a polymeric liquid, and the structure of the proposed measuring system would remain the same.

A 137Cs radioactive source (emitter energy: 0.662 MeV) and two 25.4 mm NaI transmitted photon detectors were used to detect emitted photons. The detectors were positioned at a distance of 250 mm from the source at angles of 0 and 13 degrees. It is worth mentioning that the simulated structure used in this study was validated in several experiments in our previous research [4,10].

**Figure 1:**
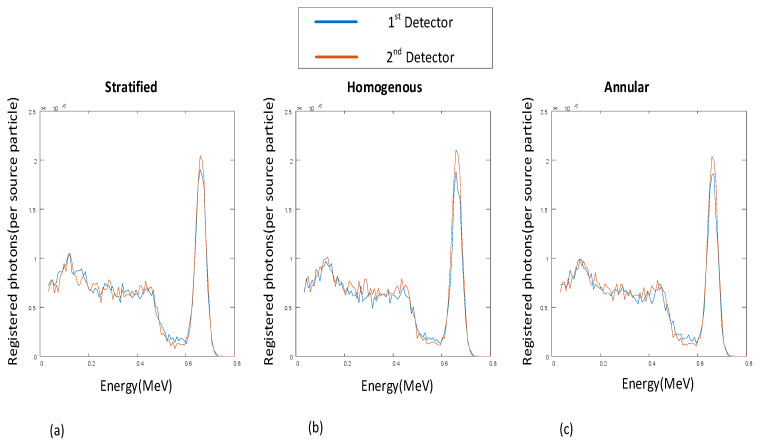
Registered signals in the 1st detector and 2nd detector (void fraction = 5%) for three flow regimes: (**a**) Stratified, (**b**) Homogenous, (**c**) Annular.

The energy spectra of the registered photons for both detectors in three flow regimes are illustrated in Figure 1.

## 3. Discrete Wavelet Transform

**Figure 2:**
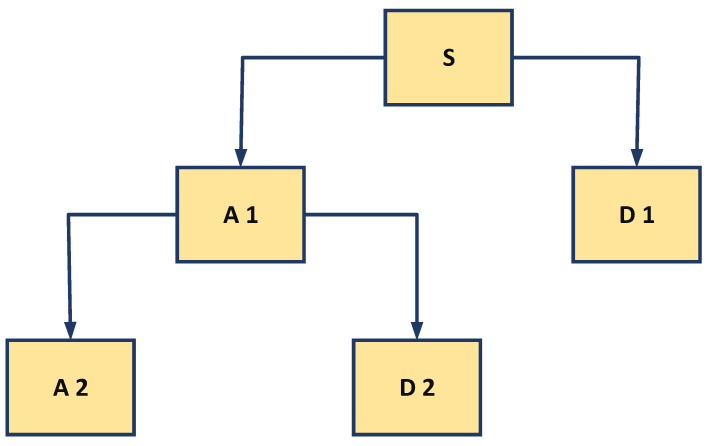
Decomposition tree of discrete wavelet transforms.

The wavelet decomposition tree is shown in Figure 2. The approximation signal in the initial stage was also divided into new approximation and detail components and the procedure was reiterated [25,26].

There are many well-known wavelet families, such as Haar, Coiflet, Symmlet, and Daubechies wavelets [27], etc., which have a wide range of applications. There is no certain way to choose a specific wavelet family in research. The choice of wavelet functions depends on the application. One of the greatest advantages of the Haar wavelet algorithm is the fact that it is easy to compute and simple to understand [28].

## 4. Feature Extraction

Feature extraction is utilized for highlighting specific patterns, with the purpose of decreasing the substantial data loss. Classification and prediction can be carried out more accurately when the pattern is represented by the principal features of the signal. Feature engineering methods can be considered as a key element in classification and prediction problems [26].

In this study, at first the registered signals of both detectors were decomposed using a discrete wavelet transform (DWT). Following this, the approximation and detail components of the signals were calculated using the Haar wavelet family. Finally, the statistical features of the approximation signal were extracted [26] utilizing the average value (*m*), kurtosis (*g*), standard deviation (σ), and RMS. Formulations relating to these features are shown in Equations (1)–(4), respectively:

(1) $$m=\frac{1}{N}\sum_{n=1}^{N}x\left[n\right]$$

(2) $$g=\frac{m_{4}}{\delta^{4}},\;m_{4}=\frac{1}{N}\sum_{n=1}^{N}{\left(x\left[n\right]-m\right)}^{4}$$

(3) $$\sigma =\sqrt{\frac{1}{N-1}\sum_{n=1}^{N}{\left(x\left[n\right]-m\right)}^{2}}$$

(4) $$\mathrm{RMS}=\sqrt{\frac{1}{N}\sum_{n=1}^{N}{\left|X\left[n\right]\right|}^{2}}$$

**Figure 3:**
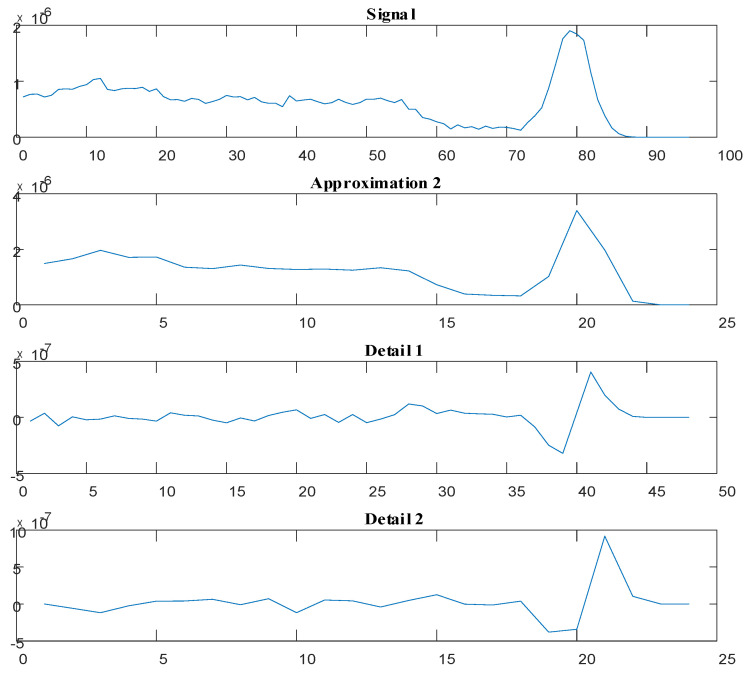
Wavelet transform of the first detector’s signal for the annular regime with a void fraction of 5%.

The wavelet transform of the first detector’s signal (annular) is shown in Figure 3.

### 4.1. Extracting Same Features from Both Detectors

**Figure 4:**
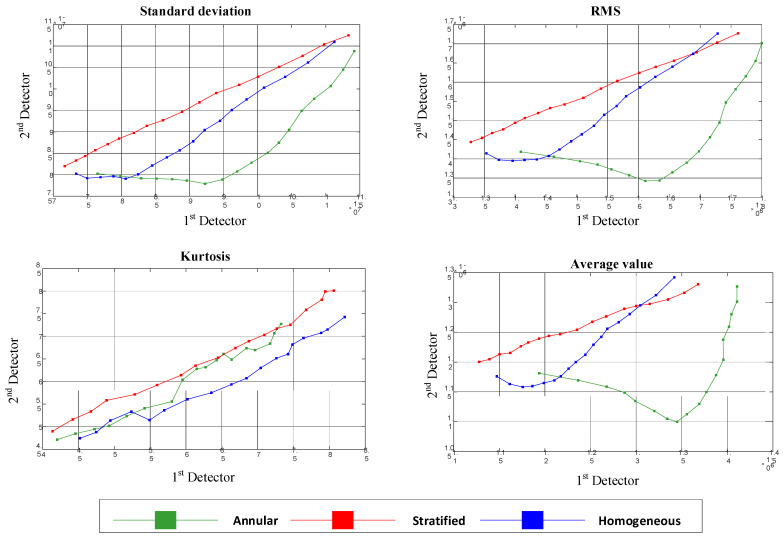
The same features extracted from both detectors.

In the first case, the same features were extracted from the approximation signals of both detectors. In Figure 4, the diagram of the extracted features from the first detector versus the second detector is shown, illustrating the separation ability of each feature.

As a result of the overlap of the illustrated features in Figure 4, the flow regimes could not be classified using these features.

It should be noted that the points illustrated in each graph are related to different void fraction percentages.

### 4.2. Extracting Different Features from Both Detectors

**Figure 5:**
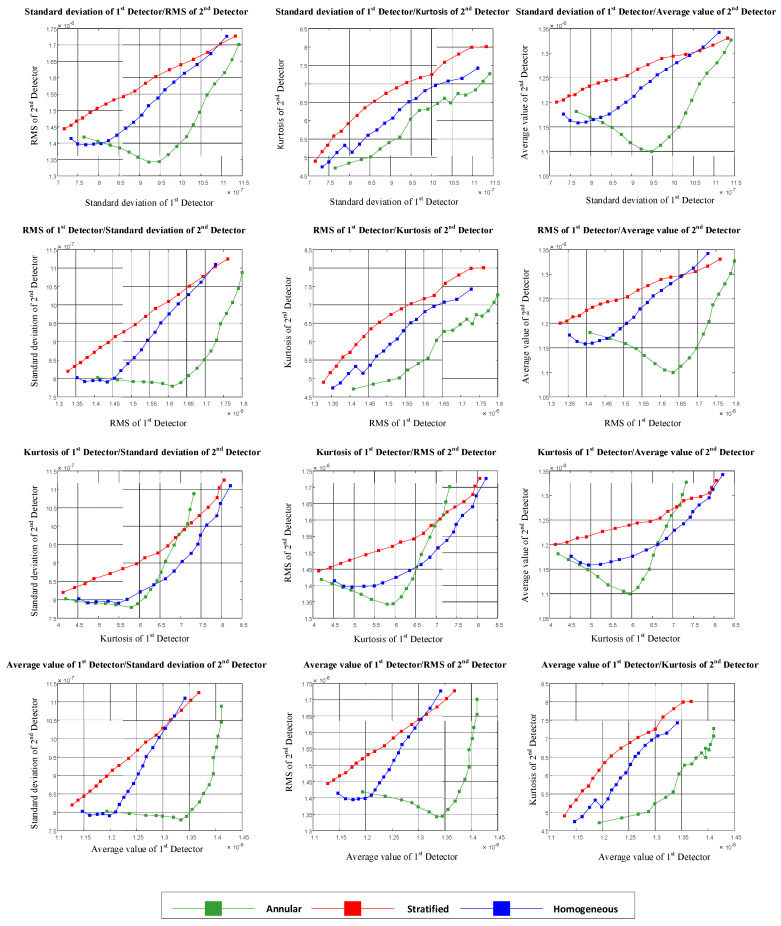
Different features extracted from each detector.

In this section, different features of the approximation signal were extracted from each detector. The diagram of the extracted features from the first detector versus the second detector is shown in Figure 5, illustrating the separation ability of each feature.

## 5. Feature Selection

**Figure 6:**
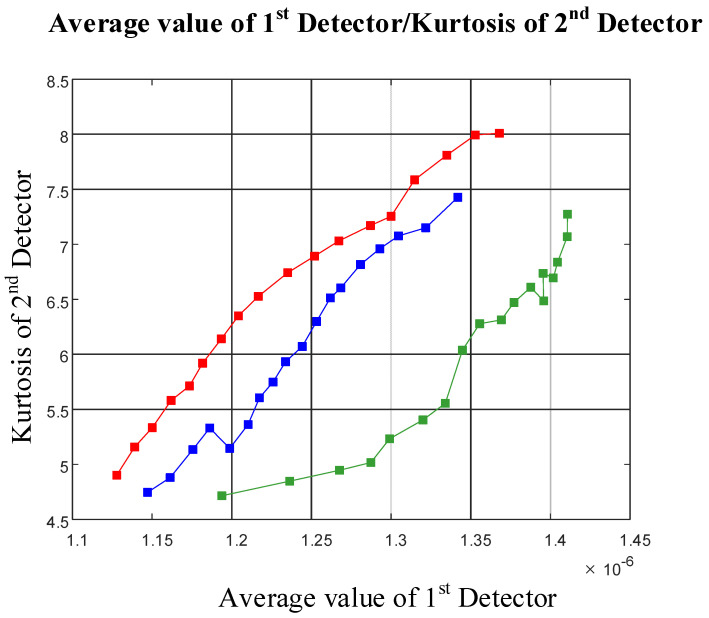
One of the features with separated graphs.

**Figure 7:**
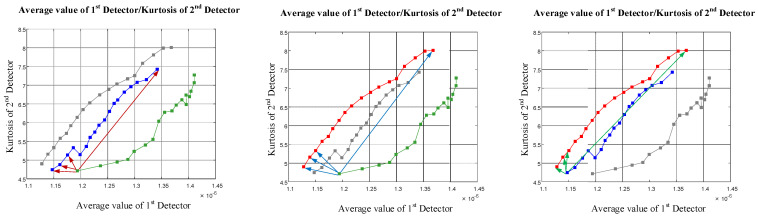
The calculation method for the SA parameter.

Considering Figure 4, it is possible to recognize type of flow regime using three features (the standard deviation of the approximation signal of the first detector versus the kurtosis of the approximation signal of the second detector, the RMS of the approximation signal of the first detector versus the kurtosis of the approximation signal of the second detector, and the average value of the approximation signal of the first detector versus the kurtosis of the approximation signal of the second detector). For investigating the separation ability of these three features, a novel parameter called the SA parameter, where SA stands for separation ability, was calculated as shown below. One of the three mentioned features is shown in Figure 6 for analyzing the operation of the SA parameter. The calculation methods for the SA parameter are shown in Figure 7 and Equations (5) to (8).

(5) $$D1=\frac{1}{N}\sum_{i=1}^{n}\sum_{j=1}^{n}\sqrt{{\left(x_{ai}-x_{hj}\right)}^{2}+{\left(y_{ai}-y_{hj}\right)}^{2}}$$

(6) $$D2=\frac{1}{N}\sum_{i=1}^{n}\sum_{j=1}^{n}\sqrt{{\left(x_{ai}-x_{sj}\right)}^{2}+{\left(y_{ai}-y_{sj}\right)}^{2}}$$

(7) $$D3=\frac{1}{N}\sum_{i=1}^{n}\sum_{j=1}^{n}\sqrt{{\left(x_{hi}-x_{sj}\right)}^{2}+{\left(y_{hi}-y_{sj}\right)}^{2}}$$

(8) $$\mathrm{SA}=\frac{D1+D2+D3}{3}$$

where D1, D2, and D3 in the equations are the average distances between the annular– homogeneous, annular–stratified, and homogeneous–stratified regimes, respectively.

SA stands for separation ability.

By calculating the SA parameter for all three separated cases, the average value of the approximation signal of the first detector and the kurtosis of the approximation signal of the second detector can be recognized as the features that are able to classify the three flow regimes, and can be applied as inputs to the ANN, aiming to enable flow regime classification and void fraction measurement.

## 6. Artificial Neural Network

In the past few decades, various advanced computational approaches, e.g., finite element, numerical linear algebra, statistics, numerical analysis, tensor analysis, and artificial intelligence, have been applied in various fields of study such as chemical engineering [29,30,31,32,33,34,35,36,37], electrical engineering [38,39,40,41,42,43,44,45,46], biomedical engineering [47,48,49,50,51,52,53,54], civil engineering [55,56,57,58], social sciences [59,60,61,62,63,64,65,66,67,68,69], mechanical engineering [70,71,72,73,74,75,76,77], computer and information technology engineering [78,79,80,81], physics [82,83,84,85,86,87], petroleum engineering [88,89,90,91,92,93,94], mathematics [95,96,97,98,99,100], etc. The ANN has been demonstrated to be the most potent technique for classification and prediction among the aforementioned computational methods. The ANN is an appropriate tool for handling modeling, optimization, prediction, and classification. These networks are mathematical systems made of simple processing elements called neurons, with parallel performance in single or multiple layers. One of the most well-known and widely used neural networks is the multilayer perceptron (MLP). The idea behind artificial neural networks comes from biological neural networks.

In this study, two different networks were utilized for recognizing the three flow regimes and measuring the void fraction percentages. The average value of the approximation signal of the first detector and the kurtosis of the approximation signal of the second detector were used as the inputs for both networks. A total of 54 different cases in three different regimes were simulated using MCNPX code. A dataset containing 39 cases (about 70%) was used for training the network and a dataset of 15 cases (about 30%) was used for evaluating the network.

**Figure 8:**
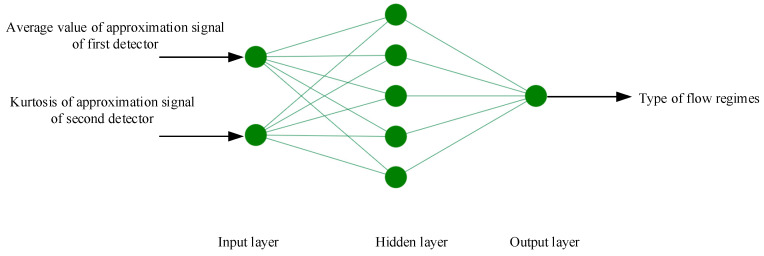
Network structure for regime classification.

Several networks with different parameters were analyzed, which led to finding the optimized network. The network characteristics and the diagram of the optimized network for regime classification are shown in Table 1 and Figure 8, respectively.

**Figure 9:**
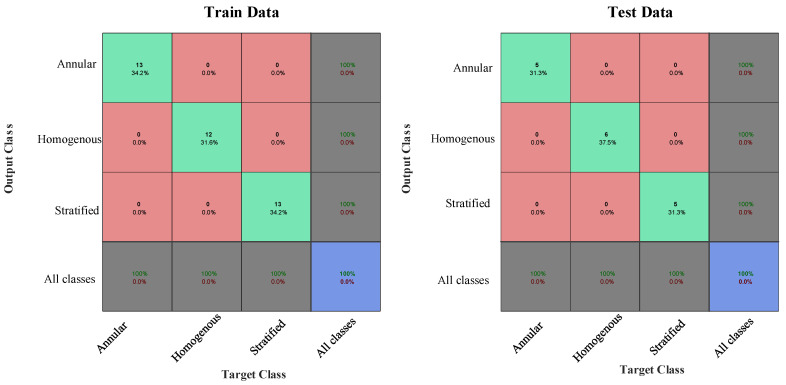
Confusion matrix for regime classification (training and testing data samples).

The performance of the obtained neural network for classifying the flow regimes is shown in Figure 9.

According to the presented results (Figure 9), with the aid of the proposed techniques, all the considered regimes were identified correctly.

**Figure 10:**
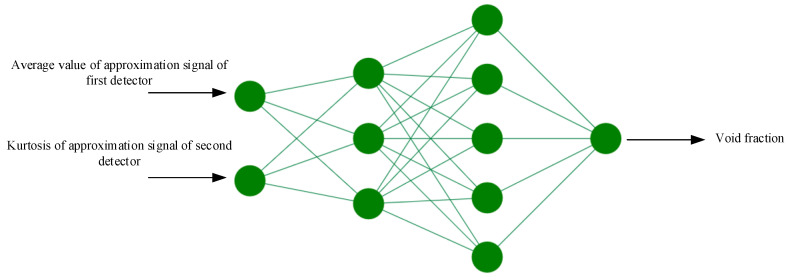
Network structure for predicting volumetric percentages.

In the second network, the same inputs were applied to the ANN, and the output of the network was the percentage void fraction. The characteristics of the neural network utilized to predict the void fraction are shown in Table 2 and Figure 10.

**Figure 11:**
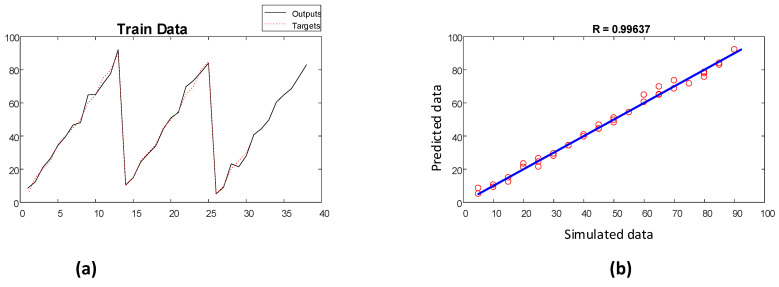
Performance of void fraction estimator network (training): (**a**) fitting diagram and (**b**) regression diagram.

**Figure 12:**
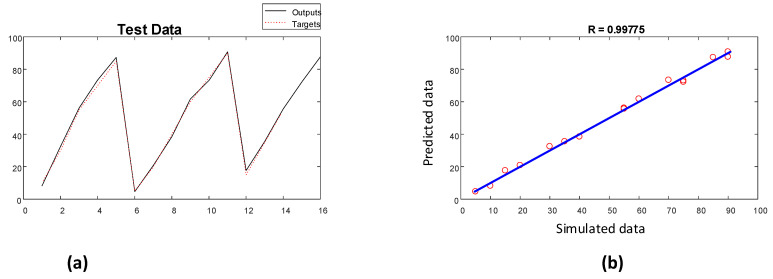
Performance of void fraction estimator network (testing): (**a**) fitting diagram and (**b**) regression diagram.

The performance of the implemented network for determining the void fractions of the training and testing datasets is illustrated in Figure 11 and Figure 12, respectively.

The obtained errors of the proposed artificial neural network model are shown in Table 3, where the MRE% and RMSE values were calculated using Equations (9) and (10), respectively.

(9) $$\mathrm{MRE}=\frac{1}{N}\sum_{j=1}^{N}\left|\frac{X_{j}(Sim)-X_{j}(Pred)}{X_{j}(Pred)}\right|$$

(10) $$\mathrm{RMSE}={\left(\frac{\sum_{j=1}^{N}{\left(X_{j}(Sim)-X_{j}(Pred)\right)}^{2}}{N}\right)}^{0.5}$$

where *N*, *X* (*Sim*) and *X* (*Pred*) stand for the number of data points and the simulated and predicted values of the neural network, respectively. The low error for the testing set validates the presented model and also shows that overfitting has not occurred.

Table 4 shows a comparison between this study and other relevant studies in this field.

Measuring volumetric percentages is a matter of key importance in many industries. Dozens of investigations have been carried out in this field to accomplish this task more accurately and decrease the rate of errors. According to the information shown in Table 4, the current study shows better performance and a lower rate of errors. The three studies mentioned adopted time-domain as well as statistical features to calculate volumetric percentages. In this study, by utilizing wavelet feature extraction, all the flow regimes were classified correctly, and the calculated void fraction percentages had notably lower error rates in comparison with the previous studies.

The simulated and predicted values of the implemented network for void fraction percentages can be found in Table 5.

## 7. Conclusions

To conclude, three conventional flow regimes were simulated using MCNP code. The output signals of both transmitted photon detectors were decomposed using a discrete wavelet transform. Several statistical features were extracted from the approximation signals of both detectors. All these features were analyzed in order to find the best features with the highest ability to classify the relevant flow regimes, for use as the inputs to the networks. The average value of the approximation signal of the first detector and the kurtosis of the approximation signal of the second detector were found to be the features with the best separation ability. One ANN was used for classifying the three flow regimes and another ANN was employed for predicting the void fraction percentages. The low error rates of the presented ANNs demonstrate the precision and correctness of the presented models.

It should be noted that in the current research, using the described methods, all the flow regimes were identified correctly, and the volume fraction percentages were determined with an RMSE of less than 1.93, which is about three times better than in previous work [4].

## Figures and Tables

**Table 1:** Network characteristics for regime classification.

Input Layer	2 Neurons
First hidden layer	5 neurons
Output layer	1 neuron
Epochs	250
Activation function	Tansig

**Table 2:** Network characteristics for void fraction measurement.

Input Layer	2 Neurons
First hidden layer	3 neurons
Second hidden layer	5 neurons
Output layer	1 neuron
Number of epochs	300
Activation function	Tansig

**Table 3:** Errors related to training and testing procedures.

Data Set	MRE%	RMSE
Training	0.33826	1.7886
Testing	0.40168	1.9227

**Table 4:** Comparison between current study and previous research.

Refs	Predicted Volume Fractions (RMSE)
[10]	2.12
[14]	5.32
[101]	6.12
Current study	1.92

**Table 5:** Simulated and predicted values for volumetric percentages.

Simulated	Predicted Values Using ANN
10	8.1759
30	32.4837
55	56.1648
70	73.3724
85	87.3543
5	4.6828
20	20.8134
40	38.4591
60	61.7835
75	73.1175
90	90.8015
15	17.6312
35	35.4866
55	55.5997
75	72.1979
90	87.6535

## Data Availability

Data is contained within the article.
